# Moderation of Services’ EKC through Transportation Competitiveness: PQR Model in Global Prospective

**DOI:** 10.3390/ijerph20010293

**Published:** 2022-12-24

**Authors:** Muhammad Shahzad Sardar, Nabila Asghar, Mubbasher Munir, Reda Alhajj, Hafeez ur Rehman

**Affiliations:** 1 Department of Economics and Statistics, University of Management and Technology, Lahore 54770, Pakistan; 2 Department of Economics, Division of Management and Administrative Science, University of Education Lahore, Lahore 54770, Pakistan; 3 School of Engineering and Natural Sciences, Istanbul Medipol University, Istanbul 34810, Turkey; 4 Department of Computer Science, University of Calgary, Calgary, AB T2N 1N4, Canada; 5 Department of Health Informatics, University of Southern Denmark, 5230 Odense, Denmark

**Keywords:** transportation competitiveness, services’ EKC, carbon emissions, institutional quality, population density, penal quantile regression model

## Abstract

The continuously increasing GHG emissions have created environmental pollution and several challenges to ecosystems and biodiversity. The challenges of climate change are multipronged, resulting in melting glaciers, flash floods, and severe heat waves. In this regard, the adaptive and mitigation strategies to manage the consequences of climate change are highly important. The transport sector creates a quarter of carbon emissions, and this share is continuously increasing. Accordingly, this research study uses transport competitiveness to determine carbon emissions of the transport sector for 121 countries covering the time period from 2008 to 2018. The Panel Quantile Regression (PQR) technique is engaged to analyze the study results. The findings highlight that transport competitiveness tends to increase carbon emissions of the transport sector across quantile groups 1 and 3, while it reduces carbon emissions in quantile group 2. The U-shaped services’ EKC is validated in quantile groups 2 and 4. The moderation engaged, i.e., transportation competitiveness, changes the turning point of the services’ EKC across quantile groups 2 and 4. However, in the high-CO_2_ quantile group, the moderation impact of transport competitiveness is strongest as it reduces the sensitivity by flattening the services’ EKC. Furthermore, the planned expansion of the population and improved institutional quality tend to mitigate carbon emissions across different quantile groups. The policy relevance/implications that are based on the study results/findings are made part of the research paper.

## 1. Introduction

The transport sector is essential as it facilitates the mobility of people and the trade of goods, which creates economic development [1,2,3]. The backward and forward linkages of the transportation sector connected with different economic sectors generate employment and encourage economic growth [4,5,6]. Despite the merits of the transportation sector in promoting economic growth, it consumes a significant share of fossil fuels and generates 25 percent of the total Greenhouse Gas (GHG) emissions [3,7,8]. The increased globalization and rapidly growing urbanization have increased the use of fossil fuels in the transport sector, substantially growing carbon emissions over time [2,3,6]. The rising GHG emissions of the transportation sector have dire consequences as it creates several environmental, health, and food security issues [3,9,10,11,12,13].

The transport sector includes different modes of transportation such as road, rail, sea, and air transportation [3,14]. These different modes of transportation are pre-requisites for the delivery of traded goods, which plays a central role in economic growth. For instance, road transportation is essential for agricultural and industrial growth as roads facilitate the transportation of raw material to agricultural farms/industrial units [14,15]. Similarly, rail transportation ensures the supply of raw material and finished goods to agricultural farms/industrial units for speedy production processes. Sea and air transportation is frequently used for international trade, which promotes the benefits of absolute/comparative advantage [3,15,16,17]. The majority of the different types of transportation uses fossil fuels, which releases carbon emissions. However, the transport sector creates almost a quarter of total emissions and plays a significant role in environmental pollution. Furthermore, the high rate of services sector growth is also triggering the expansion of the transport sector, and it is expected that carbon emissions of the transport sector will continue to grow in the future [3,17]. The rising GHG emissions have increased the world mean temperature levels beyond the desired threshold levels and poses serious environmental threats to natural habitats. Furthermore, a large number of animal and plant species are endangered from the adverse impacts of climate change [3,14,15,17].

The rapidly growing emissions released by the transport sector motivated researchers/policymakers to think about the environmental sustainability of this sector in multiple ways [3,18,19,20]. One way is to introduce renewable fuel in the transport sector, and the second is to incentivize this sector to develop zero-emissions green technologies [3,20,21,22,23]. In this regard, some countries of the world have agreed to manufacture electric cars with net-zero emissions up to 2050 [3,20,23]. The regulation of energy prices, tax rebates for promoting green technologies, recycling, and carbon taxing have helped the environmental sustainability of the transport sector across different countries [24,25,26,27]. The sustainability of the transportation sector requires capacity-building of institutions, corruption-free governance, and the very coordinated interaction of the transport sector with motivation to mitigate carbon emissions [20,22,28].

In the recent past, electric vehicles (EVs) have gained tremendous popularity on several grounds as compared to the Internal Combustion Engine (ICE) [17,29,30]. The ICE uses fossil fuels and directly contributes to environmental pollution [29,31]. EVs have a certain advantage of renewable energy use, which helps achieve net-zero emissions targets. However, the pace of production and usage of EVs is a little slower due to various reasons such as higher production cost and slower consumer adoptability [17,29,31]. The limited distance traveled by EVs on one charge is also a major hurdle in the slow growth of EVs. Furthermore, the slower expansion of EV utilization infrastructure is a significant hurdle in the slow adoption of EVs in the majority of countries [29,30]. In the context of international bindings, it is predicted that 100 million electric vehicles are expected to be on the road by 2035. Furthermore, predictions have also been made by different agencies to raise global EV production to 548 million by 2040 [17,29,31]. Charging stations also need to be modernized for quick charging to save the time of EV users. International standardization is also required for the manufacturing of EVs and charging stations [17,29,30].

The concept of carbon neutrality is very important due to the increasing use of fossil fuels, which ultimately increases carbon emissions [32,33]. The concepts of carbon neutrality and biocapacity are interlinked as they refer to environmental sustainability [33,34]. The leading factors that include addressing carbon neutrality includes the adoption of various carbon mitigation strategies such as the use of renewable fuels in production processes and the promotion of green technologies in the transport sector [32,34]. Furthermore, carbon taxing and rebates on the usage of environmental production technologies provide financial incentives to mitigate carbon emissions [33,34,35].

In the last 20 years, the services sector’s share in the World’s Gross Domestic Product (GDP) has increased from 50 percent to 64 percent, indicating that this sector has grown significantly (World Development Indicators, World Bank). The services sector cannot be graded as a zero-emissions sector, as it is mostly services, such as tourism and transportation, that use fossil fuels, creating carbon emissions [36,37,38]. Furthermore, the process of supply chains used to deliver goods also uses different transportation facilities, which ultimately tends to create carbon emissions [36,37,39]. The embodied carbon emissions are also observed in the services sector as most of the developed countries transfer their production technologies to developing countries [3,20,28,39]. The backward and forward linkages of the services sector with other sectors create a pull effect that significantly impacts the environment [36,37,39]. The services sector is the second-largest carbon footprint creator in the economy of China [36,39,40].

This research study uses transport competitiveness to determine carbon emissions of the transport sector, while competitiveness related to the transport sector indicates the expansion of the transport sector and enhancement of quality of the transport sector [3,17]. The improvement in quality of the transport sector is inclined to increase cost efficiencies and the adoption of green technologies for carbon mitigations, because a broader concept of transport competitiveness is used to determine the carbon emissions of the transport sector [41,42]. However, the literature indicates that different countries are adopting different policies to promote EVs by reducing petrol/diesel-fuel-based vehicles. Furthermore, a significant portion of emissions, i.e., above 90 percent, is based on road transportation, which frequently uses fossil fuels and plays a significant role in creating urban pollution [17,42,43,44]. It is obvious that EVs do not emit carbon emissions, but it is necessary to examine, during various phases of EVs, how much emissions are reported. In this regard, different studies have generated evidence that EVs generate lesser emissions during their complete life cycle process as compared to other gasoline-fuel-based vehicles [30,42,43,45]. 

The recent assessment report of the Intergovernmental Panel on Climate Change (IPCC) expressed that human actions to promote economic growth are responsible for Greenhouse Gas (GHG) emissions [3,21,22,23]. The report further highlights that rising GHG emissions are responsible for environmental pollution, raising the Earth’s temperature and causing climate change. The transport sector emits 25 percent of global GHG emissions [3]. The World Economic Forum (WEF) records an index related to transport competitiveness by executing a managerial survey annually for more than 150 countries. The transportation competitiveness index measures the two aspects related to the transportation sector. First, it assesses the extensiveness of different means of transportation used by a country, and secondly, transportation competitiveness considers the quality aspect of different means of transportation. Therefore, this study focuses on exploring the role of transportation competitiveness to determine the transportation sector’s carbon emissions. To our knowledge, transport competitiveness is not used in research to explain the variations in the transport sector’s emissions with the services sector.

This research fills the existing research gap in several ways. First, this research study engages the role of the services sector to determine the services’ Environmental Kuznets Curve (EKC) based on the emissions of the transport sector. Secondly, the study analyzes how transport sector moderation impacts its emissions. Thirdly, the study highlights the dynamics of changes in the services’ EKC by engaging a moderator (transportation competitiveness). Furthermore, this research study also incorporates the impact of institutional strengthening and population expansion to determine the transport sector’s carbon emissions. The theoretical grounds of the research study are based on two famous environmental models known as the Environmental Kuznets Curve (EKC) and the Stochastic Impact by Regression on Population, Affluence and Technology (STIRPAT) framework [46,47,48,49,50,51,52].

The three important research objectives to meet the identified research gaps are as follows:

1. To ascertain the impact of transport competitiveness in determining the transport sector’s emissions;
2. To empirically confirm the services-based EKC for transport sector emissions;
3. To envisage the impact of a moderator (transport competitiveness) in ascertaining the changes in turning points and steepening/flattening of the services’ EKC.

The sequence of the remaining research study includes literature of the related studies, which is highlighted in the second section of the research paper. The theoretical framework and research methodology are presented in the third and fourth sections, respectively. The results/discussions regarding the analysis are portrayed in the fourth section of this research paper. The conclusions/policy implications based on the analyzed results are depicted in the fifth section.

## 2. Literature Review

An updated review of the literature provides insights about the research topic. In this regard, an attempt is made to ascertain an effective literature review by covering different dynamics of the research study. Accordingly, the literature review section includes recent papers validating the EKC and STIRPAT model, followed by relevant studies regarding the moderation of carbon emissions.

Simon Kuznets advocated the theory of the EKC in 1955 to explain the behavior of income growth and inequality. Subsequently, this theory was tested in the field of environmental economics by a large number of researchers on different datasets [13,47,49,50,51,52]. The literature further indicates that the theory of the EKC is validated for its different shapes. In this regard, the inverted U-shaped EKC was confirmed for different countries by taking different datasets [3,53,54,55,56,57]. Similarly, the U-shaped EKC was confirmed by several researchers for various datasets [3,13,58,59,60,61,62]. The sectoral-based EKC for different sectors was validated by different researchers [3,63,64,65,66,67]. The literature also indicates that some researchers have used service value addition to validate the EKC [13,62,67].

The Stochastic Impact by Regression on Population, Affluence and Technology (STIRPAT) is employed in environmental analysis to ascertain the role of various variables on the environment [48,52,62,68,69,70]. In the STIRPAT model, carbon emissions are frequently used as a dependent variable for environmental pollution [8,18,71,72]. The population is a necessary part of the STIRPAT framework to determine environmental pollution as planned population expansion mitigates carbon emissions [55,61,62,73]. Some research studies have also been found use the service value addition to determine the sectoral impact on environmental pollution [13,62]. Institutional quality is an important determinant of carbon emissions, as improved institutional quality tends to lessen emissions of the transport sector [74,75,76,77], and poor institutional quality increases carbon emissions [3,78,79,80].

In environmental economics, different variables are used as moderators for observing changes in income and emissions. In this regard, the moderation of energy consumption and financial development of the EKC for Turkey was used, and the moderation of financial development has resulted in a stronger emergence of the inverted U-type EKC [81]. In the case of BRICS countries, a moderating role of corruption in determining the income-induced carbon emissions was observed. The study results indicated that controlling corruption could positively mitigate income-based carbon emissions [82]. The moderation of R&D investment for income-induced emissions in thirty provinces of China was observed, and the study found that R&D investment has a significant role in mitigating income-based carbon emissions by promoting green/renewable technologies in different provinces of China [83].

The moderation of renewable energy for foreign direct investment and GDP in the case of Pakistan was engaged. The findings of the research study elaborated that foreign direct investment and GDP growth tend to increase carbon emissions when moderated by renewable energy consumption [84]. The impact of financial development for Malaysia was assessed for moderating the EKC. The study results indicated that the EKC is not confirmed, while financial development significantly moderates income-induced carbon emissions [85]. Energy consumption was used as a moderator for the GDP growth of middle-income countries, and the moderation of energy consumption is observed for economic development in reducing carbon emissions [86]. The moderation of financial development was engaged for 115 countries to validate the EKC across different income groups of countries. The study results regarding the moderation of financial development revealed the observance of stronger EKCs across different income groups [87]. The effect/moderation of urbanization for validation of the EKC was used in the case of Turkey, and the study results indicated that the moderation of urbanization was observed in an inverted U-shaped EKC [88]. The moderation of natural resource dependence for determining the EKC of Malaysia was used, and the study results indicated that the moderation of natural resources impacts income-based EKCs [89].

Researchers used the data from 31 African countries to envisage the moderation of tourism for economic growth, and the study results revealed that tourism further exacerbates economic-growth-based carbon emissions [90]. The moderation of globalization on energy consumption, financial growth, economic development, and human capital for emissions of G20 countries was assessed. The study results indicated that moderation of globalization positively impacted the role of HR and financial growth for carbon emissions [91]. The moderation of technology and its composition of EKC in the case of global countries was assessed [92]. The moderation of financial growth for different indicators such as economic development, energy use, and urban development in determining the carbon emissions of Turkey was assessed [93]. A study conducted to ascertain the impact/moderation of reusable energy and HR for GHG emissions revealed that the moderation/impact of reusable energy for pesticide usage indicates the mitigation of GHG emissions [94]. The moderation effect of institutional governance on emissions for 30 provinces of China was assessed, and the study results revealed that institutional quality weakens the economic development process, which may bring an early peak of emissions in China [95].

The literature review of different research studies established the research/study gap that the competitiveness of the transport sector has not been considered in determining the transport sector’s carbon emissions. The recent literature on moderating variables for carbon emissions also indicates that the role of competitiveness for the transport sector has not been considered to assess the variations in the latent mechanism of the services’ EKC. More precisely, to our knowledge, the existing literature does not indicate that competitiveness of the transport sector, as a moderator, is employed to explain variations in the EKC and carbon emissions nexus. Based on the literature and research gap, the objectives are reassembled to identify the role of competitiveness of the transport sector in ascertaining its impact on the transport sector’s emissions. The emergence of transportation-based carbon emissions and the services’ value-addition EKC is tested. Finally, the moderation/impact of competitiveness of the transport sector is considered to determine the variations in transportation and services’ value-addition nexus.

## 3. Theoretical Framework

**Figure 1:**
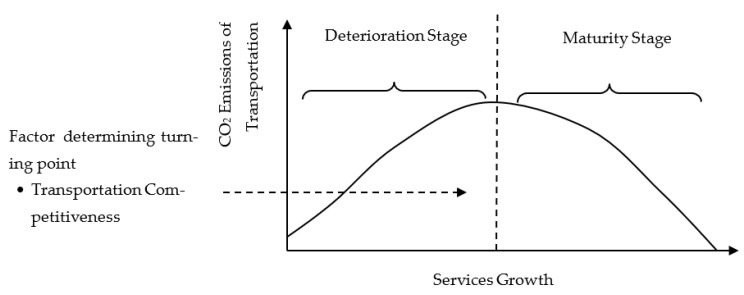
Theoretical Framework (Inverted U-Shaped EKC).

**Figure 2:**
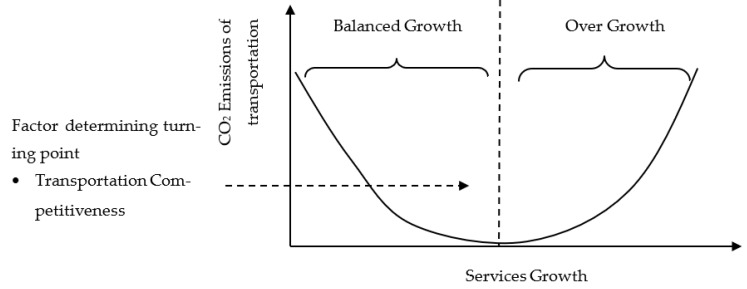
Theoretical Framework (U-shaped EKC).

To address the research goals/objectives, the theoretical framework is prepared based on the EKC and STIRPAT frameworks. In this regard, two options of the theoretical foundation are considered. The first is validating the inverted U-shaped EKC based on transportation carbon emissions and services’ value addition. Figure 1 indicates the inverted U-shaped relationship between the services sector’s value addition and transportation sector’s carbon emissions. The second possibility of the theoretical framework represents the U-shaped EKC, indicating two stages of development (Figure 2).

## 4. Materials and Methods

A comprehensive methodology is adopted considering a panel of 121 countries (Appendix A). These countries are based on the World Economic Forum (WEF)’s recorded transportation competitiveness data. The WEF recorded this transportation competitiveness data in 2008 for member countries. The study period is confined to 2018 because the COVID-19 pandemic was reported to start in 2019 and the transportation activities were suspended. The dynamic grouping of the data is prepared based on emissions of the transport sector. In this regard, four different dynamic quantile groups are prepared using annual data of emissions of the transport sector for sample countries. These categories of groups are named as low CO_2_, low medium CO_2_, high medium CO_2_, and high CO_2_. These categories/groups are prepared on the basis of actual values of emissions of the transport sector for each year. Consequently, the sample countries are expected to change/jump category/groups if they show progress over time. Furthermore, the dynamic panel grouping is made on the basis of the actual values of each country in respective time periods, and countries may change group. This grouping is better as compared to the static grouping made by the World Bank on income groups, etc., used for panel data analysis [3,96,97]. The static grouping is made on the basis of one value for a country and its previous position in which the specific country’s fall is totally ignored [17,98,99]. Furthermore, this dynamic grouping is not overlapped, as dynamic panel quantile grouping allows movement of a country from one year to another and, in this regard, a more realistic grouping can be made [3,17,96,97,98,99]. The study variables with the relevant source are presented in Table 1.

As indicated in Table 1, the dependent variable is considered as carbon emissions of the transportation sector. The data for this variable are measured in metric tons per capita and are downloaded from the Emissions Database for Global Atmospheric Research (EDGAR). The services’ value addition and square of services’ value addition are independent variables, and their data are taken from World Development Indicators (WDIs). To explain the variations in environmental pollution, several researchers have used this variable in their analysis [13,62]. Researchers in environmental analysis frequently use variables related to the population. In this research study, population density is used as one of the explanatory variables for environmental pollution in the transportation sector [55,61,62]. The high institutional quality index ensures the proper implementation of rules and regulations to mitigate carbon emissions. By keeping in view the importance of institutional quality, this variable is included in the analysis to determine carbon emissions in the transportation sector [3,74,75,76,77]. The World Economic Forum (WEF) recorded member countries’ transportation competitiveness index in 2008. WEF collects data for transportation competitiveness through the execution of a perception survey annually. The transportation competitiveness index measures the expansion of the transport sector and its quality. Considering the importance of the transportation competitiveness index, the impact/moderation of competitiveness of the transport sector is included in the model to determine the transport sector’s carbon emissions.

CO_2_(T) = f (SVC, SVC^2^, TC, POP, TEC) (1)

Based on the functional form of the research study, the standard form of the equation is as follows:

CO_2it_ = β_0_ + β_1_ SVC_it_ + β_2_ SVC^2^_it_ + β_3_ SVC_it_ × TC_it_ + β_4_ SVC^2^_it_ × TC_it_ + β_5_ TC_it_ + β_6_ PDEN_it_ + β_7_ INST_it_ + μ_it_ (2)

The non-linear term of the services sector is included in the model to empirically validate the services’ EKC based on carbon emissions of the transportation sector. For the inverted U-shaped EKC, β_1_ > 0 and β_2_ < 0 have to be significant. However, in the U-shaped EKC, β_1_ < 0 and β_2_ > 0 are significant. The moderation of transportation competitiveness is included in the model to determine the changes in the direction of the slope of the services’ EKC and its relative flattening/steepening [3,100]. To determine the changes in the direction of the slope of the EKC, we differentiate Equation (2) concerning SVC and then, by equating it to zero, we obtain:

SVC* = (−β_1_ − β_3_TC)/(2β_2_ + 2β_4_TC) (3)

Equation (3) shows that the turning point of the services’ EKC depends on the value of the moderator (transportation competitiveness). To determine the changes in the turning point, we differentiate Equation (3) concerning transportation competitiveness and obtain the following equation:

δSVC*/δTC = (β_1_ β_4_ − β_2_ β_3_)/2(β_2_ + β_4_TC)^2^ (4)

The denominator of Equation (4) is positive due to the square term. Therefore, the changes in the turning point are contingent on the numerator term (β_1_ β_4_ − β_2_ β_3_). If the numerator term, i.e., (β_1_ β_4_ − β_2_ β_3_), is positive, then the turning point will tend to the right side and vice versa. This derivation indicates that the changes in slope direction of the services’ EKC are not only contingent on β_3_ but also on β_1_, β_2_, and β_4_ [3,14,100,101]. On the contrary, the flattening or steepening of the services’ EKC is straightforward as it only depends on β_4_. The cross-product of the square term of services growth with the moderator, i.e., transportation competitiveness, indicates the flattening or steepening of the services’ EKC and depends on β_4_. If β_4_ is positive and significant, then the services’ EKC will tend toward flattening for the inverted U-shaped services’ EKC. The steepening will occur for the inverted U-shaped services’ EKC when β_4_ is negative and significant. Similarly, in the U-shaped services’ EKC, the positive and negative significance of β_4_ indicates the steepening and flattening of the services’ EKC for transportation competitiveness, respectively [3,100,101].

The Dawson method prepares graphs to validate the moderation of transportation competitiveness. The regression coefficients of services growth, square of services growth, and cross-products are used [3,102]. The graph displays the movement of the services curve at low and high levels of transportation competitiveness and represents the variations in curvilinear behavior of services growth at various levels of the transport competitiveness (moderator) [3,14].

The basic foundations of IPAT identity and the STIRPAT framework are used to derive the research study model in its logarithm form. The STIRPAT framework is derived on the basis of standard IPAT identity, which is as follows:

I_it_ = a P_it_b A_it_c T_it_d e_it_ (5)

where “I_i_” denotes the environmental impact, “P_i_” denotes population size, “A_i_” shows affluence, and “T_i_” is the technology. By using a natural log, the following standard form of the STIRPAT model is derived:

ln I_it_ = a + b (ln P_it_) + c (ln A_it_) + d (ln T_it_) + ln e_it_ (6)

It is also appropriate that once the standard STIRPAT model is derived, additional variables can also be added according to the scope and dimensions of the research study [3,13,15,70]. Accordingly, the extended STIRPAT model of the study based on the variables highlighted in Table 1 is as follows:

ln (CO_2it_) = β_0_ + β_1_ (ln SVC_it_) + β_2_ (ln SVC^2^_it_) + β_3_ (ln SVC_it_ × TC_it_) + β_4_ (ln SVC^2^_it_ × TC_it_) + β_5_ (TC_it_) + β_6_ (ln PDEN_it_) + β_7_ (INST_it_) + μ_it_ (7)

The Panel Quantile Regression (PQR) estimates the study results. The PQR estimation technique has many benefits over conventional regression estimation techniques. The PQR estimation technique uses the median as the average rather than the mean because the mean is affected by extreme values in the dataset [3]. In panel datasets having large cross-sections, it is observed that most of the variables are non-normal, but the PQR is a very suitable technique for the estimation of results having non-normal variables as it inhabits the median as a measure of average [3]. The PQR estimation procedure dates back to 1978 when Koenker and Bassett advocated for this estimation model in their seminal paper [103].

Furthermore, the PQR estimation technique is not distribution-sensitive and produces robust results [3,104,105]. The PQR model provides results based on countries considered in quantiles and may be useful in preparing a policy for the countries of quantile panels [3,96,98,99,106]. In environmental analysis, many researchers have used the PQR model to analyze the results, especially to determine the key determinants of carbon emissions [99,106]. As discussed earlier, the PQR model provides robust results when dealing with panel data having non-normal variables. The recently available literature shows that PQR is extensively engaged in analyzing panel data for considering various quantiles for envisaging environmental pollution [3,96,97,106,107].

## 5. Results and Discussion

The basic statistics included in the research study are calculated to ascertain the information regarding the concentration and dispersion of the data. The measures of average include mean/average, median, mode, and percentiles, while the indicators for dispersion are standard deviation, interquartile range, and skewness/kurtosis. The summary of these various statistics of central tendency and measures of dispersion regarding different variables is elaborated in Table 2. The variance inflation factor (VIF) is also calculated for identifying multicollinearity based on the correlation coefficient for the study variables (Table 3). VIF shows that none of the values of VIF > 10 indicate any issue of multicollinearity [3,13].

The skewness/kurtosis normality test is used to test the normality of the data series considered for analysis. The significance of this normality test for all the variables indicates that data series considered in the analysis are reportedly non-normal, and the outcomes of these test values are summarized in Table 4. As a consequence of the non-normality of data series of the variables, the Panel Quantile Regression (PQR) estimation procedure is engaged for estimation purpose as this technique is not distribution-sensitive and can generate robust results [104,105]. The analyzed results of the PQR estimation technique are portrayed in Table 5.

The study results are analyzed in four quantile categories/groups, which are developed based on transport sector emissions for a group of sample countries. These quantile groups are named based on the level of emissions of the transportation sector. The services sector’s value addition significantly increases emissions in quantile groups 1 and 3. This may indicate that the growth of the services sector in quantile groups 1 and 3 requires more transportation to commute people and goods from one place to another, increasing the transport sector’s carbon emissions. However, in quantile groups 2 and 4, the services sector significantly tends to lessen emissions of the transportation sector, indicating that services sector growth promotes transportation that has the potential to mitigate carbon emissions across the high-CO_2_-emitting group of countries [3,13,62].

The non-linear services value addition is considered in the study/research model to empirically confirm the EKC proposition [3,13,62]. The signs of the coefficients for services growth and its square term in group 1 have not changed, which shows non-confirmation of the EKC. For group 2, the signs of the coefficient of services growth and its square term have changed from negative to positive, indicating the validation of the U-shaped EKC [13,62]. The EKC proposition is not confirmed in group 3, as the square term of the services growth coefficient is insignificant. Finally, the EKC hypothesis in the U-shape is supported in group 4 as both coefficients of the services growth and its square term are significant and have negative and positive signs, respectively [13,62]. The validation of the U-shaped services’ EKC across groups 2 and 3 indicates that the services sector is growing beyond the sustainability phase, and expanding the services sector exacerbates environmental pollution. This indicates that expanding services require more commutation facilities, which further increases transport sector emissions. The non-validation of the EKC across quantile groups 1 and 3 indicates that the expansion of the services sector is not in line with sustainability principles [3,13,62].

The survey results indicate that transportation competitiveness significantly upsurges emissions of the transport sector in categories/groups 1 and 3. However, in quantile group 2, the competitiveness of the transport sector tends to lessen the transport sector’s emissions, while it reports insignificance in quantile category/group 4. The impact of competitiveness of the transport sector in generating emissions is strongest for quantile group 1, indicating that the services sector is growing, but environmental protection has not been prioritized. In quantile group 2, transportation competitiveness tends to lessen emissions of the transport sector, indicating that the quality of different modes of transportation is considered a priority to protect the environment from carbon emissions [3,13,62].

**Figure 3:**
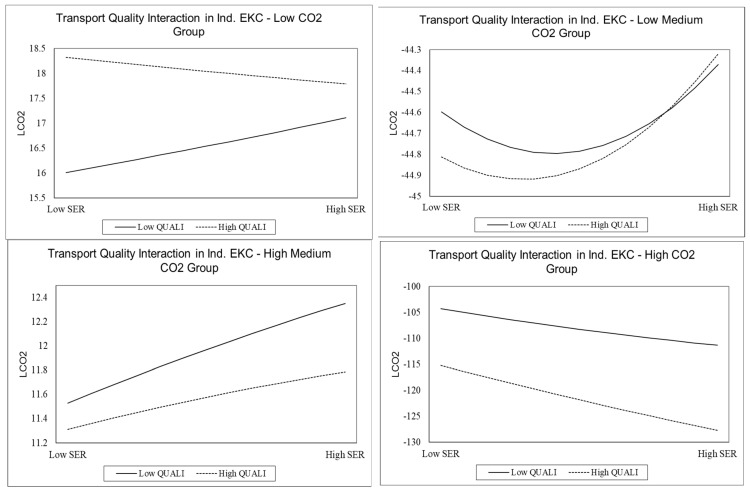
Interaction of Transportation Competitiveness for Services’ EKC.

The moderation of transportation competitiveness is also considered by including the cross-product terms. The outcomes of moderation are summarized in Table 6 and Figure 3. It is revealed that the EKC is not validated in groups 1 and 3. However, the U-shaped EKC is confirmed for quantile groups 2 and 4. In the case of quantile group 2, the moderator (transport competitiveness) shifts the turning point toward the left side and further steepens the services’ EKC. In quantile group 4, the moderator shifts the turning point toward the left side and flattens the EKC. The moderation analysis also specifies that the left side shifting of the turning point of the U-shaped services’ EKC in groups 2 and 4 shows the early maturity of the turning point. This indicates the early completion of the sustainability phase of the U-shaped services’ EKC. However, in group 4, the flattening of the U-shaped services’ EKC indicates that the moderator reduces the sensitivity of carbon emissions by the services’ sector. Group 4 has high-CO_2_-emission countries, and the most advanced countries are adopting environmental protection policies in the transportation sector. The adoption of green technologies in the transportation sector by group 4 countries has improved transportation competitiveness, implicitly moderating the services sector’s carbon emissions. The Dawson methodology used in Figure 3 also indicates the significant contribution of moderators in determining the carbon emissions across quantile group 2 and group 4. In group 4, it is evident that a higher level of transportation competitiveness at increasing services growth tends to mitigate the transportation sector’s carbon emissions. A possible explanation is adopting green technology and sustainable fuels used in the transportation sector [13,62]. 

The impact of institutional quality index and population growth/density is also included in the STIRPAT model to assess their detrimental role in explaining transport sector emissions. The institutional quality is observed to significantly lessen the transport sector emissions for quantile groups 1, 2, and 3 [74,75,76,77]. The study indicates that environmental protection policies are more efficiently pursued and implemented in the following countries. However, the institutional quality index is reported as insignificant in quantile group 4. The analysis further reveals that population density is significantly mitigating the transportation sector’s emissions across quantile groups 1, 3, and 4 [55,61,62,73]. The planned expansion of population requires lesser means of transportation for commuting from one place to another as most of the necessary amenities of life such as commercial hubs, and education and health facilities are available within the community premises, which tends to reduce the carbon footprint [3,62].

## 6. Conclusions

This research study is conducted on a sample of 121 countries to assess the moderation of transportation competitiveness in determining the transport sector’s carbon emissions. This research study’s novelty is engaging transportation competitiveness to study the services sector’s growth and transportation-based emissions nexus. This aspect has not been found during the literature review to the best of our knowledge. Three precise objectives of the research study are considered to address the research gap. First, the role of competitiveness of the transport sector is assessed in determining the environmental pollution created by the transport sector. Secondly, the empirical validation of the services’ EKC is tested, and, finally, the moderation (transportation competitiveness) is envisioned to ascertain variations in the services’ EKC. The more updated estimation approach, i.e., Panel Quantile Regression (PQR), is used. The Dawson methodology is used to confirm the moderation impact of competitiveness for the transport sector. The data of 121 sample countries are divided into four dynamic categories/groups based on emissions of the transport sector.

The competitiveness of the transport sector tends to lessen emissions in category/quantile group 2, indicating that the quality of the transport sector in this group of countries is mitigating carbon emissions. However, in quantile groups 1 and 3, expanding the transportation sector to support the services sector’s growth tends to stimulate the transport sector’s carbon emissions. The U-shaped services’ EKC is confirmed for categories/quantile groups 2 and 4, while, in groups 1 and 3, the services’ EKC is not supported. The moderation (transportation competitiveness) is analyzed for changes in the services’ EKC. The changes in the turning point of the services’ EKC across quantile groups 2 and 4 are observed to shift toward the left side. However, the non-linear moderator steepens the services’ EKC in quantile group 2 and flattens the services’ EKC in quantile group 4. Quantile group 4 is a high-CO_2_ group and mostly includes countries opting for green technologies such as electric vehicles and zero-emissions commitments. Therefore, in these countries, the quality of transportation has improved, and transportation competitiveness reduces the sensitivity of the services’ EKC by flattening its shape.

The implications of the research study are evident as the quality of different modes of transportation needs to be improved in terms of green technologies and transportation sustainability. This will improve transportation competitiveness, which will help mitigate the services’ growth leading to the transportation sector’s carbon emissions, especially across quantile groups 1 and 3. In quantile groups 1 and 3, where the services’ EKC is not validated, the sample countries need to improve transportation competitiveness to achieve the sustainability phase. The moderation of competitiveness for the transport sector is observed, elucidating increasing transportation quality to lessen carbon emissions. Furthermore, the systematic population growth and improvement in institutional quality across all quantile groups of countries may help to mitigate pollution in the transportation sector. The study results are very helpful for international donors, policymakers, urban developers, and transport planners. The research study has data limitations and a limited scope of transportation competitiveness. In the future, more variables such as renewable energy, institutional quality, and innovations can be used to moderate the transportation sector’s carbon emissions. Furthermore, other sectoral value additions may also be used to determine the emissions of the transport sector.

## Figures and Tables

**Table 1:** Study Variables.

Abbreviation of Variables	Full Name of Variables	Data Sources
CO_2_	Log of Transportation Sector CO_2_ Emissions (Mt/Capita)	EDGAR *
SVC	Log of Services Value Added	WDI **
SVC^2^	Log of Square of Services Value Added	WDI **
PDEN	Log of Population Density	WDI **
TC	Transportation Competitiveness Index	WEF ***
INST	Institutional Quality Index	ICRG ****

* Emissions Database for Global Atmospheric Research (EDGAR); ** World Development Indicators (WDI); *** World Economic Forum (WEF); **** International Country Risk Guide (ICRG).

**Table 2:** Descriptive indicators/Statistics of the Study Variables.

Variables	CO_2_	SVC	TC	INST	PDEN
N	1331	1288	1282	1282	1331
Mean	2.0040	3.9828	4.2214	4.1299	4.3050
Median (P_50_)	1.8780	4.0188	4.2217	3.9227	4.4557
SD	1.8978	0.2194	1.2030	0.8904	1.4328
IQR	2.4183	0.2621	1.8283	1.3881	1.7611
Range	10.2119	1.4858	5.2558	3.7275	8.4541
P_25_	0.7798	3.8691	3.2911	3.4180	3.4813
P_75_	3.1982	4.1312	5.1194	4.8062	5.2425
Skewness	0.1539	-0.9690	0.0868	0.5314	-0.1784
Kurtosis	2.8588	4.3175	2.1617	2.2769	3.5289

**Table 3:** VIF Matrix for Study Variables.

	CO_2_	SVC	TC	INST	PDEN
CO_2_	-	-	-	-	-
SVC	1.0870	-			
TC	1.1366	1.2745	-	-	-
INST	1.0625	1.1636	3.2059	-	-
PDEN	1.0008	1.0695	1.0303	1.0099	-

**Table 4:** Skewness/Kurtosis Tests for Normality.

Variables	Observations	Pr (Skewness)	Pr (Kurtosis)	Adj. Chi^2^	Prob. > Chi^2^
CO_2_	1331	0.0219	0.2983	6.31	0.0426
SVC	1288	0.0000	0.0000	-	0.0000
TC	1282	0.2025	0.0000	-	0.0000
INST	1282	0.0000	0.0000	-	0.0000
PDEN	1331	0.0080	0.0011	16.12	0.0003

**Table 5:** Estimation of PQR Results for four Quantile Groups (Dependent Variable: lnCO_2_).

Variables	Quantile Category/Group-1 (Low CO_2_)		Quantile Category/Group-2 (Low Medium CO_2_)	
	Coefficient	*p*-Values	Coefficient	*p*-Values
SVC	2.5636	0.0000 *	−22.2697	0.0000 *
SVC^2^	0.2797	0.0050 *	2.7748	0.0000 *
SVC × TC	−0.7915	0.0000 *	0.1266	0.0020 *
SVC^2^ × TC	−0.0304	0.0000 *	0.0053	0.0000 *
TC	4.2621	0.0000 *	−0.6238	0.0000 *
INST	−0.3846	0.0000 *	−0.1695	0.0000 *
PDEN	−0.2107	0.0000 *	0.0178	0.2390
**Variables**	**Quantile Category/Group-3** **(High Medium CO_2_)**		**Quantile Category/Group-4** **(High CO_2_)**	
	**Coefficient**	***p*-Values**	**Coefficient**	***p*-Values**
SVC	4.3341	0.0680 ***	−45.3359	0.0000 *
SVC^2^	−0.3026	0.2230	5.6323	0.0000 *
SVC × TC	−0.2903	0.0080 *	0.5913	0.0000 *
SVC^2^ × TC	0.0086	0.0000 *	−0.0504	0.0000 *
TC	0.8563	0.0510 ***	−0.5256	0.4160
INST	−0.0295	0.0140 **	0.1125	0.0220 **
PDEN	−0.0997	0.0000 *	−0.0929	0.0000 *

*, **, and *** show levels of significance at 1%, 5%, and 10%, respectively.

**Table 6:** Moderation (Transportation Competitiveness) for Observing Changes in EKC.

Quantile Groups	Category	Shape of EKC	Changes in Turning Point	Flattening/Steepening
Group-1	Low CO_2_	Not Validated	-	-
Group-2	Low Medium CO_2_	U-Shaped	Left	Steepening
Group-3	High Medium CO_2_	Not Validated	-	-
Group-4	High CO_2_	U-Shaped	Left	Flattening

## Data Availability

The following datasets are available in the given link https://databank.worldbank.org/source/world-development-indicators (accessed on 20 December 2021).

## Appendix A

**Table A1:** Sample of Four Quantile Groups Based on Carbon Emissions of Transportation Sector.

Category/Group-1 (Low CO_2_)	Category/Group-2 (Low Medium CO_2_)	Category/Group-3 (High Medium CO_2_)	Category/Group-4 (High CO_2_)
Armenia	Albania	Austria	Algeria
Barbados	Azerbaijan	Azerbaijan	Argentina
Bhutan	Bahrain	Bangladesh	Australia
Botswana	Bangladesh	Belgium	Belgium
Brunei Darussalam	Benin	Bolivia	Brazil
Burundi	Bolivia	Bulgaria	Canada
Cambodia	Bosnia and Herzegovina	Colombia	Chile
Chad	Botswana	Chile	China
Cyprus	Cambodia	Czech Republic	Colombia
Estonia	Cameroon	Denmark	France
Gabon	Costa Rica	Ecuador	Germany
Georgia	Croatia	Ethiopia	Greece
Guinea	Cyprus	Finland	India
Haiti	Dominican Republic	Ghana	Indonesia
Iceland	El Salvador	Greece	Italy
Jamaica	Estonia	Guatemala	Japan
Lesotho	Ethiopia	Hungary	Malaysia
Liberia	Georgia	Ireland	Mexico
Madagascar	Ghana	Israel	Netherlands
Malawi	Guatemala	Jordan	Nigeria
Mali	Honduras	Kazakhstan	Pakistan
Malta	Jordan	Kenya	Philippines
Mauritania	Kenya	Kuwait	Poland
Mauritius	Latvia	Luxembourg	Russia
Mongolia	Lebanon	Morocco	Saudi Arabia
Mozambique	Lithuania	New Zealand	South Africa
Namibia	Luxembourg	Nigeria	Spain
Nepal	Mongolia	Norway	Thailand
Nicaragua	Mozambique	Oman	Turkey
Rwanda	Namibia	Paraguay	Ukraine
Senegal	Nepal	Peru	UAE
Seychelles	Nicaragua	Philippines	UK
Sierra Leone	Panama	Portugal	US
Tajikistan	Paraguay	Qatar	
Uganda	Senegal	Romania	
Zambia	Singapore	Singapore	
Zimbabwe	Slovenia	Sri Lanka	
	Sri Lanka	Sweden	
	Tajikistan	Switzerland	
	Trinidad and Tobago	Tunisia	
	Tunisia	Ukraine	
	Uruguay		
	Zimbabwe		
37 countries (24% of total sample)	43 countries (29% of total sample)	41 countries (26% of total sample)	33 countries (21% of total sample)
